# Characterizing landscape fragmentation of Koitobos river sub-basin, Trans-Nzoia, Kenya

**DOI:** 10.1016/j.heliyon.2024.e29237

**Published:** 2024-04-04

**Authors:** Kennedy Wekesa Murunga, Maurice Nyadawa, Joseph Sang, Charles Cheruiyot

**Affiliations:** a Pan African University, Institute for Basic Sciences, Technology and Innovation [PAUISTI], P.O BOX 62000-00200, Nairobi, Kenya; b Department of Civil Engineering and Construction Management, Jaramogi Oginga Odinga University of Science and Technology [JOOUST], P. O. Box: 210-40601, Bondo, Kenya; c Soil, Water and Environmental Engineering Department, Jomo Kenyatta University of Agriculture and Technology [JKUAT], P.O. Box: 62000-00200, Nairobi, Kenya; d Department of Civil, Construction & Environmental Engineering, Jomo Kenyatta University of Agriculture and Technology [JKUAT], P.O. Box: 62000-00200, Nairobi, Kenya

**Keywords:** Landscape fragmentation fragmentation indices, Watershed heterogeneity, Land-use policy

## Abstract

The changes in landscape structure and functioning due to unprecedented human interference are hastening across the globe and it is thus a compelling necessity to preserve and restore our ecosystems. This study aimed to characterize levels of landscape fragmentation, habitat structure, driving forces, and perceptions of the residents on the most preferred reconfiguration approaches. The land use/land cover [LULC] change was first determined by interpreting the 1973, 1986, 1995, 2002, 2014, and 2022 Landsat images using the QGIS 3.26 while the selected landscape fragmentation metrics were analyzed using FRAGSTATS 4.2. Forests, shrubs, and grasslands showed a declining trend, except for agriculture, water, and built-up areas, which depicted high increases for the study periods [1973 to 2022]. The landscape of the study area is characterized as progressively fragmenting as signified by high escalated values of patch number [374 %], edge density [7828 %] between 1986 and 2002, contagion [10.3%], and a declined value of Shannon Diversity Index [SHDI] [-17.42%], Shannon evenness index [SHEI] [-25.8 %] and connectedness [-43.3%]. Considering these results, high losses of forests and grasslands coupled with expansive farmlands and built-up areas have led to unprecedented landscape fragmentation From field surveys and oral interviews, this has not only left streams vulnerable to massive sediment loads but has also triggered annual floods which occur during wet months even though change in onset of rainfall seasons was also reported. The findings call for restoration and integrated and sustainable restoration efforts, especially for the forests, grasslands, and riparian corridors along with sustainable urban planning and community-based sensitization on watershed management.

## 1 Introduction

Increasing human activity has profoundly impacted the natural environment [1], notably evidenced by landscape fragmentation, which detrimentally affects ecosystems and biodiversity [2]. Landscape fragmentation entails segmenting large land cover areas into smaller isolated patches, a phenomenon influenced by human actions and natural alterations in land use and cover [3]. The expansion of human populations has spurred various activities including urbanization, agricultural expansion, infrastructure development, and cultural encroachment, further exacerbating landscape fragmentation [[3], [4], [5], [6], [7]].

Landscape refers to diverse geographical areas consisting of interacting ecosystems and human actions [8,9]. In recent decades of the 20th century, shifts in land use patterns due to human activities have become a significant factor influencing landscape structure [[1], [10]], configuration, and dynamics [2,11]. The primary concern revolves around landscape fragmentation stemming from various human interventions such as infrastructure development [12], mining, population growth, deforestation [13], agricultural expansion, and fulfilling human necessities [14,15]. These alterations result in the degradation of natural ecosystem functions [16], increased surface runoff leading to flooding, changes in water quantity and quality, climate variations, habitat loss, and demographic shifts within the landscape [9,17,18].

Landscape fragmentation varies over time and space [19]. For example, floodplains experience greater variability in land use and land cover compared to mountainous areas and islands, primarily due to their vulnerability to relatively straightforward modifications [20]. [20,21]. Alterations in land use and land cover can result from human activities and natural calamities [[22], [23], [24]]. Landscapes across the world are heavily disturbed and transformed [2]. Research has shown that about 32% of landscapes across the world have been altered, particularly between 1960 and 2019 [3,1].

Concerns regarding the repercussions of altering land use patterns due to deforestation and agricultural expansion or abandonment have led to a crisis concerning the quality of water and soil resources [4]. Given that economic and human activities primarily occur at the landscape level, it is deemed an appropriate spatial scale for examining environmental changes resulting from human activities over an extended period [25]. Consequently, evaluating landscape changes and assessing historical human land use serve as dynamic tools for sustainable land use planning [5].

In this context, landscape metrics serve as algorithms for quantifying the spatial characteristics of patches, classes, or mosaics across the entire terrestrial landscape [26]. Landscape metrics offer a valuable means to compare the landscape states of various land uses [27,28]. The utilization of landscape metrics is crucial in distinguishing the distinct attributes of different land use types from one another. Furthermore, they aid in better monitoring the impact of land use changes on hydrological processes [[29], [30]] and nutrient cycles [9]. Thus, understanding the impacts of human activities across various sectors or land use types, serving as primary data in spatial-temporal landscape analysis, holds particular significance for interpreting and modeling land changes, as well as comprehending the relationships between environmental and human factors [27].

Landscape fragmentation stands out as a pivotal process illustrating the influence of human activities on the disruption of land structure and function [3]. This process involves the division of the landscape into smaller patches, signifying a transformation tailored for human utilization [31], which detrimentally impacts biodiversity. Notably, it emerges as a significant consequence of land degradation, diminishing crucial ecosystem connections [32]and habitat corridors across the landscape [[33], [34], [35]]. Analyzing landscape fragmentation holds the potential to inform strategic development approaches aimed at enhancing land restoration and conservation efforts [15,36]. Landscape fragmentation, stemming from shifts in land use and land cover, facilitates altering different land use and cover categories. As a result, such fragmentation may diminish ecological diversity, productivity, functional capacity, connectivity, and overall coherence [[37], [38], [39]].

The impacts of landscape fragmentation are influenced by intensive land-use practices, which affect qualitative and quantitative components of land-covers [17,18]. The rapidly increasing human population, which has triggered an ever-growing demand for food, wood products, and energy, is one of the primary drivers of land-use, land-cover [LULC]changes, and thus landscape fragmentation and habitat loss. Further, the rate and extent of land cover conversion and unprecedented human modification of environments beyond recovery levels have triggered changes in ecosystem functioning [19,20].

Various studies have confirmed that anthropogenic landscape fragmentation results in habitat loss and ecosystem services [40] offered by different habitats such as forests, shrubs, grasslands, wetlands, and water bodies [[5], [6], [7]]. Disturbance and alteration of natural habitats of any magnitude affect the physical attributes of ecosystem processes and the environment, a scenario that ultimately results in ecological degradation [8]. Further, studies have revealed that landscape fragmentation leads to changes in temperature [41] within watersheds while also affecting the hydrological processes [[11], [12], [29], [30]].

The rate of anthropogenic landscape degradation is increasing worldwide, especially in the African regions where a rapid human population is being experienced [21,22]. Further, ecosystems in the East African region are frequently restructuring due to complex societal and biophysical factors [23]. For example [24], revealed that fragmentation in East Africa is reflected by the increase in croplands by 34.8 % for the period 1998–2017 while 20 million hectares of woodlands had been converted to less woody classes due to fragmentation [42]. LULC due to the rocketing human population, fragmented watershed governance, and livestock keeping, is a crucial and usual occurrence in heterogeneous watershed areas of Kenya [43,1].

Although understanding the impacts of human activities on natural landscapes and their functioning poses a significant challenge, there exist various methodologies for collecting, storing, analyzing, and interpreting natural resource data [21]. Geospatial technologies offer a promising solution for cost-effective and rapid data collection and analysis [[21], [44]]. FRAGSTATS, a standalone software program, is specifically designed for computing a diverse range of landscape metrics to assess landscape fragmentation [[26], [45], [46]]. While numerous landscape metrics have been developed to quantify fragmentation, not all are suitable for a particular landscape analysis due to high correlations and redundancy among them [[47], [48]]. Therefore, a careful selection of metrics is essential to avoid redundancy. These metrics can measure various aspects such as area, shape, core area, nearest neighbor distances, isolation, and connectedness at patch, class, or landscape levels [27,45,49].

Previous research has demonstrated the utility of this tool in illustrating spatiotemporal dynamics of landscape change [3,[29], [30], [41], [50]].

Various studies have been carried out to assess the rate and degree of LULC change in Kenyan ecosystems and water towers [[25], [27], [28], [33], [34]]. The findings of this research indicate that, while there is a notable spatial and temporal growth of farmlands, grazing areas, and barren areas, the majority of watersheds are characterized by a reduction in ecosystems such as forests, shrublands, grasslands, wetlands, and water bodies.

Increasing human population, intensive livestock keeping, inappropriate land-use practices, ineffective land tenure laws and poor watershed governance have led to land fragmentation [35]. The situation has been exuberated by Politics, and regime change [51] that has come with different policies and enforcement [52], particularly on access, management of protected forests [36]. Moreover, maps of protected ecosystems are influenced by political power which in turn leads to the destruction of major ecosystems [53].

Koitobos River Sub-basin [KRSB] is one of the biophysical and ecological hotspots where landscape fragmentation and land cover modification have steadily occurred over the past years [35,38]. Even after the devolution of landscape-related protection functions to counties, the protection of key ecosystems is challenged by limits of institutional fix and veto players [54]. KRSB represents part of Mt. Elgon National Game Park which is home to various small, and large bird species, and mammals with a complex floristic composition [55]. KRSB is unique concerning its vegetative landscape structures [56]which are strongly defined by altitude, climate, and soil distribution [57,58]. The sub-basin is characterized by large-scale African Development Corporation [ADC], Panocal International Limited and Kenya Seed Farms [59]. KRSB also hosts major urban centers; Kitale town, Endebess, and Kwanza centers which are Trans-Nzoia headquarters and Sub-county centers respectively [60]. KRSB is crucial as it forms part of the upper drainage system that drains into Nzoia River which then flows to transboundary Lake Victoria. However, it is impacted by rapid population growth, excessive settlements and encroachment to protected areas of Mt. Elgon National Park [61], illegal logging, livestock rearing, a myriad of institutional failures, and watershed governance issues. As a result, previously large habitats are heavily fragmented while small-sized habitats are lost.

Many studies on landscape fragmentation in watersheds have been conducted at small spatial scales with individual ecosystems or fragments being considered as units of study. However, to draw inferences and conclusions about the consequences of landscape fragmentation, it is key to compare how the whole landscape has differed in its structure and patterns of fragmentation at various spatiotemporal scales [62]. Landscape fragmentation particularly structural characteristics of LULC at classes, patches and landscape levels within the Kenyan landscapes has not to a significant level received appropriate research attention. Many studies have focused on LULC changes [[42], [63], [64]] while those focused on landscape structure coupled with LULC changes in heterogeneous watersheds comprising of protected areas, highly intensive agricultural lands, human settlements, and livestock are scarce. Hence, the main objective of this research was to investigate spatiotemporal landscape fragmentation structural changes for the years 1973, 1986, 1995, 2002, 2014 and 2022 in Koitobos River sub-basin. Specific objectives included 1) To determine spatio-temporal landscape fragmentation metrics and structure; and 2) to explore the driving factors of landscape structure changes in KRSB.

The rationale of carrying out Landscape fragmentation in Koitobos Subbasin at class, patch and landscape level is to comprehensively understand its spatial structure, ecological implications, and management needs. Class metrics offer insights into the composition of different land cover types, patch metrics provide details on the configuration and spatial distribution of these patches, while landscape metrics offer a holistic view of landscape-level fragmentation patterns. By integrating these metrics, researchers can pinpoint specific areas of concern, identify drivers of fragmentation, and inform targeted conservation and land management strategies to mitigate ecological impacts and promote landscape resilience within the Koitobos Subbasin.

## 2 Materials and methods

### 2.1 Study area

Koitobos River sub-basin is located in western part of Trans-Nzoia County, Kenya between Latitude 1,0′00″ to 1,11′00″ and Longitudes 34,40′00″ and 34,90′00’’. Elevation of the subbasin varies between 1792 m above the mean sea level to 4221 m in the upper parts. It has rich natural resources such as mixed forests [Eucalyptus, bamboo trees] [65]. Maize is the recorded as the main crop in the watershed. Other crops include short term cereals [wheat, sorghum and millet], horticultural crops [tomatoes, Irish potatoes, kales, cabbages] and legumes [cowpeas and beans] [66]. The upper part of the subbasin covers part of Mt. Elgon National Park which is a protected area for mammals and flora. Trans Nzoia County is considered the food basket of Kenya due to its suitable climate for agriculture. The subbasin is drained by Koitobos river which drains an area of approximately 825 sq.km starting from Mt Elgon. It discharges into Nzoia River a few kilometers from.

**Fig. 1:**
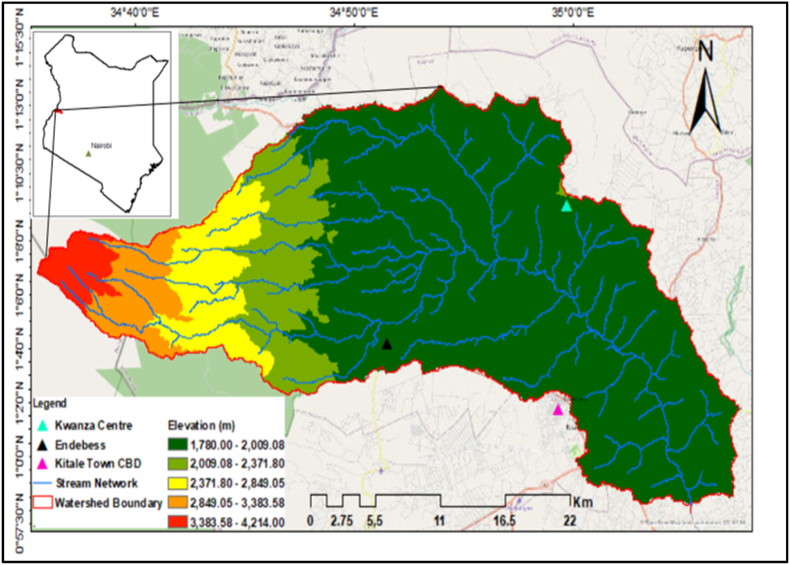
Study area.

Kitale town, which is the headquarter of Trans Nzoia County. The river is joined by small tributaries such as Muberi, and Kaibei which originate from North-Western slopes of Mt. Elgon [67]. In the middle and lower reaches, it is joined by other intermittent tributaries that drain wetland areas such as Sikubu and Chemususu. during the wet seasons; Chemususu and Sikubu wetland areas. The location of the study area is presented in Fig. 1.

**Fig. 2:**
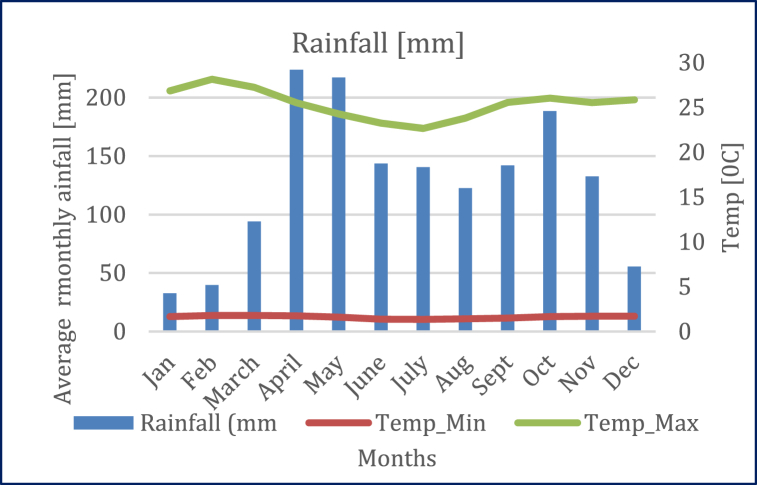
Mean monthly rainfall and temperature of Trans-Nzoia, where KRSB is located.

KRSB experiences a bimodal rainfall pattern. The rain season starts in March to end of May [MAM]. This is followed by a short rainfall period [July–September] while December–March is a dry period [35]. The rains in this sub-basin are influenced by hydrological dynamics in Mt. Elgon in the West and Cheranganyi hills in the North-East [67]. The foot-hill of Mt. Elgon experiences more annual average rainfall of about 1270 mm than the lowland areas which experiences annual average rainfall of about 1016 mm due to differences in attitude. However, a maximum of 1549 mm has ever been recorded [67]. The wet season is characterized by night temperatures of up to 19 °C rising to about 25.6 °C during the day. The temporal characteristics of rainfall and temperature is shown in Fig. 2.

Soils in the Upper parts of KRSB are Ferralsols, which are weathered soils characterized by low nutrient levels [68]. The Ferralsols are interspersed with deep and red Nitisols soils containing some good percentage of organic matter [68]. The basin has several urban centers though most of the population resides in rural areas. Most farms in KRSB are privately owned and the sizes have been decreasing to smaller sizes due to land tenure rights that have driven subdivision of land amongst family. Large corporations that own large-scale farms; Agricultural Development Corporation [ADC], Kenya Seed Company, Kenya Seed Driers, Western Seed and Kenya Cooperative Creameries, and Panacol Flower International Limited. Urban centers in this watershed include; Endebess and Kitale Town. The sub-basin also hosts part of Mt. Elgon Game Park and Mt. Elgon National Reserve.

There is an increasing emergence of urban centers in the subbasin particularly after devolution in 2013, even though most of the population resides in rural areas. Urban centers in this watershed include; Endebess and Kitale Town. The sub-basin also hosts part of Mt. Elgon Game Park and Mt. Elgon National Reserve. Most farms in KRSB are privately owned and the sizes have been decreasing to smaller sizes due to land tenure rights that have driven subdivision of land amongst family members. Large corporations that own large-scale farms; Agricultural Development Corporation [ADC], Kenya Seed Company, Kenya Seed Driers, Western Seed, Kenya Cooperative.

### 2.2 Data acquisition, and image processing

Ortho-rectified Landsat imagery of 1973, 1986, 1995, 2002, 2014 and 2022 were downloaded from the United States Geological Survey portal at https://earthexplorer.usgs.gov/and detailed information is given in Table 1. A Landsat of 1973 had a different spatial resolution of 60 m and thus, it was resampled to 30 m to ensure consistency and comparability. The selected bands for processing composites and classification were blue, green, red and the Near Infrared [NIR] bands. Calibration of the top of image atmosphere [TOA] was carried out on multi-temporal images to obtain reflectance values that were free from biasing factors and to ensure results do not originate from different sun angles [69,70]. The images were obtained during the months of March, April, May and June to ensure agricultural crop could also be analyzed.

**Table 1:** Landsat satellite Imagery Properties used for LULC classification.

Satellite and sensor	Path/Row	Date of acquisition	Resolution
Landsat 1–5 MSS	182/59	May 20, 1973	60 m
Landsat 5 TM	170/59	June 22, 1986	30 m
Landsat 5 TM	170/59	02 April 95	30 m
Landsat 7 ETM on	170/59	May 15, 2002	30 m
Landsat 8 OLI	170/59	March 05, 2014	30 m
Landsat 9 OLI	170/59	April 04, 2022	30 m

The ground truthing polygons and points of different land use and land covers were collected using the Garmin Etrex 32x GPS tool following standard procedures [[71], [72]]. Secondary data such as census data has been used to describe landscape fragmentation.

### 2.3 Land-use and land-cover [LULC] classification

Land-use and Land-Cover maps were prepared by using Supervised classification techniques [73] based on the training samples and using the maximum likelihood algorithm that has been used in many studies [[34], [73]] because of its simplicity and robustness. Training samples are polygons of known areas employed to classify the remaining part of the imagery following procedures by Refs. [74,75]. LULC classes were defined and prepared based on FAO LULC classification guidelines [76] and procedures [77].

An accuracy assessment was carried out to measure the accuracy of classified maps against the reference points obtained from the field. A total of 180 ground truthing points (30 points for each LULC category) were obtained through the stratified random sampling technique and were used for accuracy assessment. Under this process, pixels classified under different various LULC polygons were compared with ground truthing points using the error matrix [[78], [79]]. Error matrices were determined to inform of user accuracy, producer accuracy, and overall accuracy [[34], [70], [80]] and Kappa statistics [[78], [79], [81]]. Previous studies provides the description of computation methods and the meaning of accuracy measures [78,[79], [81], [82]]. Producer and user accuracies should be over 60% [83]. However, in principle some studies have suggested that the overall accuracy should meet the minimum value of 85% [72]. The Kappa coefficient of 1 implies a perfect agreement between the classified map and the reference map [84]. classified imagery which meets the accuracy criteria outlined in the Andersen classification scheme [72] can be adopted for further analysis and has been used by many researchers [2,85]. The percentage of LULC change rates and extent were calculated using Equations (1), (2)) based on the procedures outlined by Ref. [34].

(1) $$\%\;of\;change\;extent=\frac{Area\;of\;final\;year-Area\;of\;intiial\;year}{Area\;of\;initial\;year}\;x\;100$$

(2) $$\%\;rate\;of\;change=\frac{Area\;of\;final\;year-Area\;of\;intiial\;year}{Time}\;x\;100$$

**Fig. 3:**
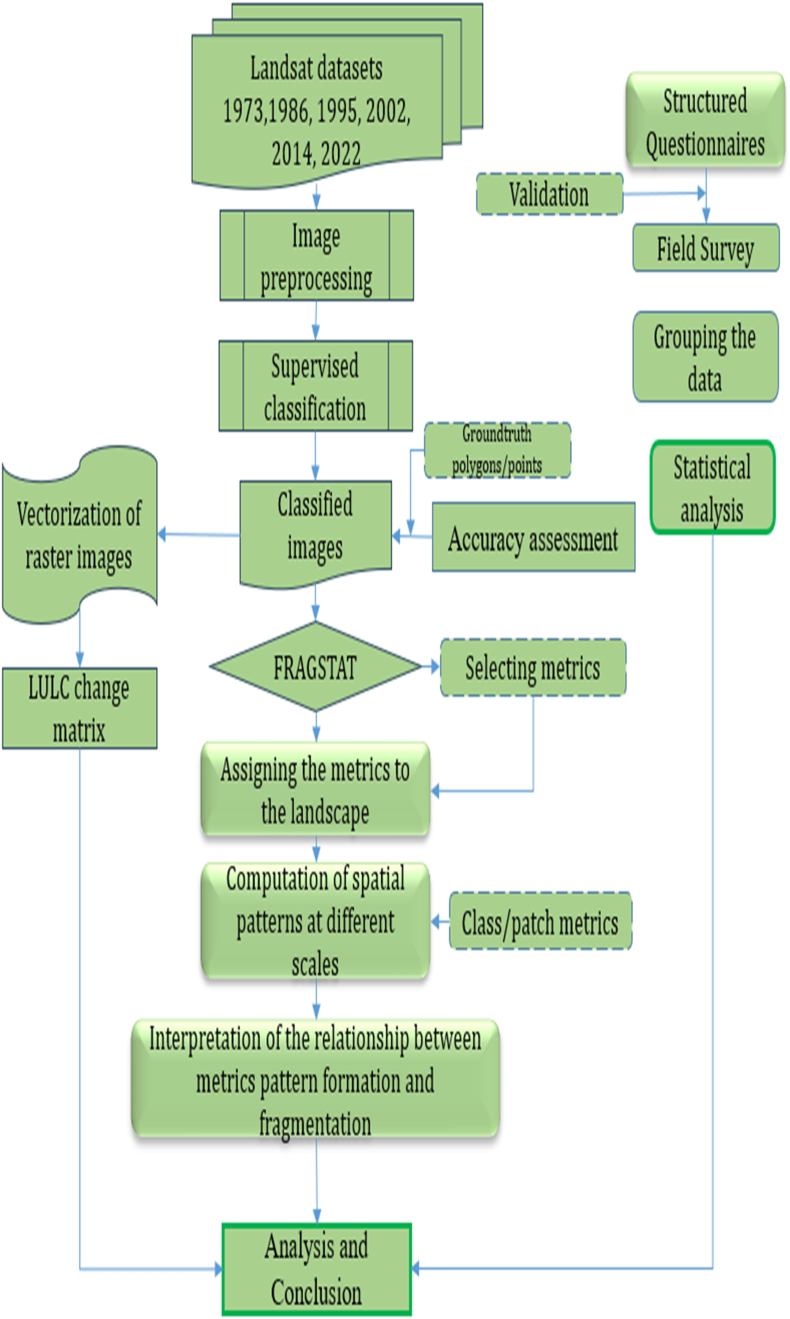
Flowchart showing steps and procedures to produce LULC maps and analysis of landscape fragmentation.

Description of selected Land Use and Land Cover for classification are presented in Table 2 while Land use/land cover classification, landscape fragmentation analysis and field survey procedures are as shown in Fig. 3.

**Table 2:** Description of selected LULC Classes.

LULC Classes	Description
Forest	Includes native forests, bamboo trees, mixed forests, and forest plantations established in Mt. Elgon in the 1990s
Shrubs	Includes bushlands consisting of small to medium woodlands
Grasslands	Includes grazing lands and areas under permanent grass cover
Agriculture	It includes crops, irrigated land, plantations, heterogeneous agricultural areas, and agro-forestry areas.
Water	Includes wetlands, swamp areas, established water storage infrastructures, etc
Agriculture	It includes crops, irrigated land, plantations, heterogeneous agricultural areas, and agro-forestry areas.
Water	Includes wetlands, swamp areas, established water storage infrastructures, etc
Built-up	It includes urban centers, roads, greenhouses, etc.

A total of 6 LULC classes were selected as they are the dominant LULC classes.

### 2.4 Quantification of landscape fragmentation

Landscape metrics under three main categories [landscape configuration and composition metrics] were extracted using procedures outlined by Refs. [[26], [47]]. In the first category, class metrics of patch number [PN], patch density [PD], Edge density [ED], interspersion and juxtaposition [IJI], connectedness, PAFRAC, and core area were considered. In the second category, patch metrics of the patch area, Euclidean Nearest Neighbor [ENN], FRAC, and contagion were considered. Average and small metrics under this category were not considered as it was determined that the smallest were limited by the spatial resolution of the satellite while the average was affected by the compromised smallest metrics. Lastly, the third category of landscape metrics included Edge density [ED], Contagion [Contag], Connectiveness, Euclidean Nearest Neighbor Distance [ENN], Interspersion and juxtaposition Index [IJI], Shannon Diversity Index [SHDI] and Shannon Evenness Index [SHI] were considered. Landscape Ecology Statistical tool [LECOS] a plugin in QGIS by Ref [86] alongside FRAGSTATS tool version 4.2.1 was used for landscape pattern analysis [47]. To achieve this, an 8-cell neighborhood rule was used to define the patches [48]. Selected metrics are described in Table 3.

**Table 3:** Description of selected landscape metrics.

Metrics	Description	Scale	Units
PAFRAC	Degree of patch complexity	Class	none
Number of patches - NP	Total number of patches in this class. Indicates the degree of sub-division	Class/landscape	none
Patch Density	Degree of landscape heterogeneity and fragmentation	Class/Landscape	No per 100 ha
Core Area	Area of interior habitat	Class/Landscape	Hectare
Edge Density	Perimeter-Area ratio	Class/Landscape	Metre/ha
Connectiveness	Functional joining between total patches of the corresponding patch within the specified distance	Class/landscape	%
Interspersion & Juxtaposition Index	Measure of patch adjacencies evenness. 100 represents 100 even and 0 for unevenness	Class/Landscape	%
FRAC	Levels of patch complexity, scale dependent. Based on patch area and perimeter	Patch	ratio
Contagion	Irregularity of patches	Patch	%
Euclidean Nearest Neighbor Distance	Edge-edge distance between neighboring patches of the same category	Patch	m
Shannon diversity index	Degree of landscape heterogeneity and diversity	Landscape	None [ratio]
Shannon evenness index	Landscape composition and richness	Landscape	None [ratio]

### 2.5 Assessing drivers of landscape fragmentation

Major diving causes of landscape fragmentation in the area were collected through key informant interviews using structure questionnaires and field observations. Random sampling followed procedures outlined by Ref. [87] while validation and validation followed guidelines by Refs. [[88], [89]]. 10 questionnaires were used to test for reliability and validity while 40 questionnaires were administered and 32 were collected [80%]. Further, oral interviews with selected locals were conducted to triangulate information from other interviewees and to establish some of the causes of landscape fragmentation. Key informants were selected based on age and experience on LULC distribution in the area [such as old farmers], responsibilities [foresters, community elders, natural resources experts], and spatial distribution/cultural representation [by altitude and resources]. This exercise followed procedures outlined by Ref. [90].

## 3 Results and discussion

### 3.1 Land use/land cover changes

**Fig. 4:**
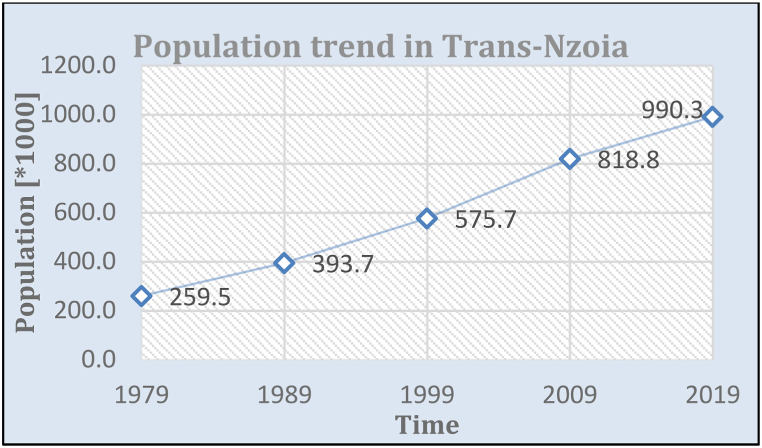
Population dynamics in Trans Nzoia where KRSB is located Modified from https://www.citypopulation.de/en/kenya/admin/rift_valley/26Trans_Nzoia/.

**Fig. 5:**
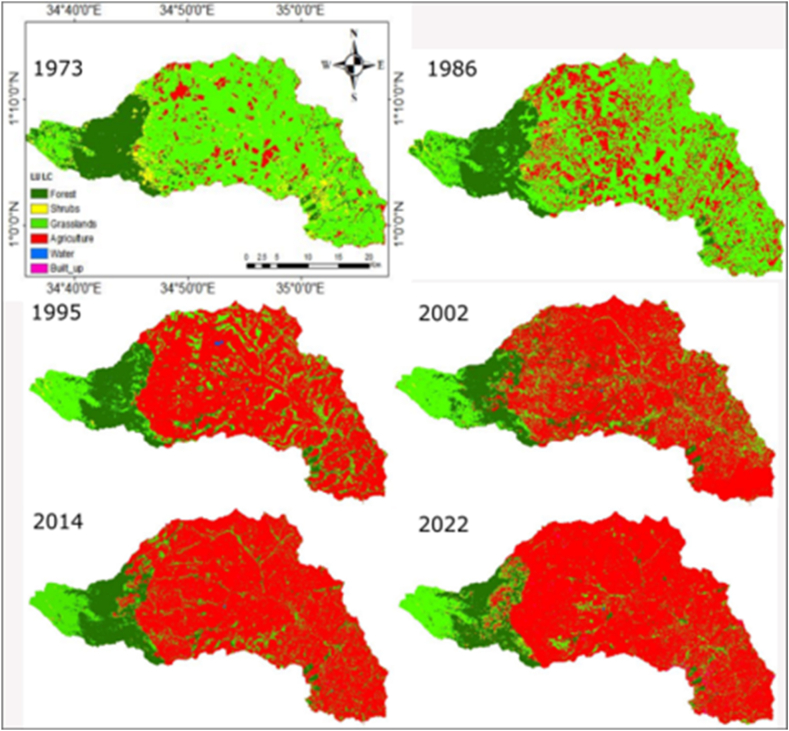
Classified LULC images for the study.

The analysis of LULC changes in the KRSB was made from 1973 to 2022 for 49 years. Forests shrubs and wetlands depicted an overall decreasing trend in area and extent, except the farmlands and built-up areas which show an increasing trend. [Fig. 5]. Forested lands, shrubs, and grasslands were dominant for the 1973 to 1986 period while farmlands and water occupied the least area. However, since 2002, farmlands and built-up areas were dominant with more isolated forests remaining in along the riparian corridors, near Kitale town and Mt. Elgon National Park. These were, a decreasing trend for forests, shrubs, and grasslands and an increasing trend for built-up areas and farmlands respectively. Farmlands increased by 56186.19 ha at an annual rate of 1146.7 ha, water for 11.25 ha [0.22 ha per annum], for the study period while built-up areas increased by 567 0.27 ha from 12.42 ha at an annual rate of 56.3 ha for the period 2002–2022. This is at the expense of forests, shrubs, and grasslands which reduced over the study period at a rate of −118.8 ha, −104.1 ha, and 935.8 ha. However, the water extent shows an increasing extent for the period 1973 to 1995 and a decreasing trend between 1995 and 2022. This indicates an ongoing conversion of natural to human-dominated ecosystems. A rapidly increasing human population, demand for wood products triggered by urbanization, increasing demand for food, and intensified agricultural activities with the perception of making maximum profits are the main factors influencing LULC changes. These findings echo similar results reported in Upper Nzoia by Ref. [[1], [91]]. Mt. Elgon ecosystems by Ref. [65] and Maasai Mara by Ref. [80]. Human population growth in Trans Nzoia County in which most of the watershed lies is depicted in Fig. 4. Overall accuracies for 1973, 1986, 1995, 2002, 2014, and 2022 classified images were 0.898, 0.95.2, 0.902, 0.915, 90.8 and 0.918. with kappa coefficients of 0.816, 0.916, 0.817, 0.835, 0.807 and 0.815 respectively. Accuracies indicate the reliability of LULC change detection maps produced. LULC maps analyzed for landscape fragmentation are presented in Fig. 5.

The increase in agricultural land and built-up area aligns with the increase in human population within the watershed. Human population growth in Trans Nzoia County in which most of the watershed lies is depicted in Fig. 4. Population in Trans-Nzoia increased at a rate of 5.1% (1979–1989), 4.6% (1989–1999), 4.2 % (1999–2009) and 2.09% (2009–2019) [Fig. 4]. Higher population increase was observed for the period 1979–2009.

As depicted in Fig. 5*,* LULC analysis shows a decreasing trend for forests, shrubs, and grasslands and an increasing trend for agriculture, urban, and water covers. Different LULCs experienced higher decreasing and increasing trends. Forest decrease rates were experienced during the period 1995–2002 period (−2.09 %), shrubs for the period 2014–2022 increased by +313.97%. Grasslands reduced by (−6.15%) for the period 1986–1995 while agriculture increased by +17.44% for the period 1986–1995. On other hand water increased by +91.6% between 1986 and 1995 while built-up areas increased by +142.63% for the period 2002–2014.

**Fig. 6:**
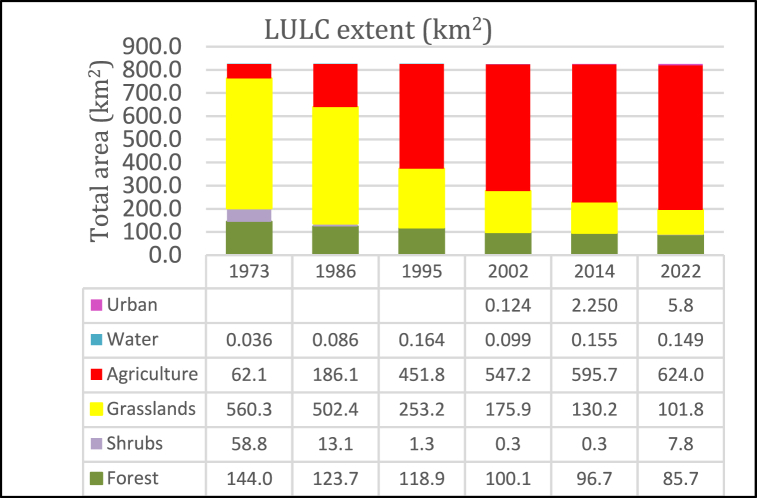
Land cover proportion km.^2^.

The lowest rates for forests were experienced between 2002 and 2014 (−0.28%), shrubs at −0.52% for the period (2002–2014), grasslands (−0.80%) between 1986 and 1995, agriculture (+0.11%) between 1995 and 2002, water (−0.509%) between 2014 and 2022 and built-up (+12.42%) between 1995 and 2002. Grasslands are the most vulnerable as they have easily been converted to agricultural land while most forests are degraded to shrubs due to an ever-increasing demand for wood products and timber for construction as a result of population increase. Built-up areas show a continuous trend especially since the County governments were established and more satellite urban centers established to serve as administrative zones and economic hubs for the increasing population. Agriculture is not only increasing but also intensifying due to population increase and high growth rates [Fig. 6]. This poses a danger of diffusive pollution to water resources especially from sediments, insecticides, pesticides, and fertilizers. Built-up areas due to urban sprawl and human settlements are on the increase and this means accelerated runoff is expected. The reduction of forests and grasslands leaves large areas without vegetation cover, making them vulnerable to erosion. This lack of vegetation also increases the generation of runoff, which can lead to flooding and soil fertility losses. Results echo the findings by Ref. [91] in upper Nzoia and [65] in the Mt. Elgon ecosystem.

### 3.2 The extent of landscape fragmentation at the class level

#### 3.2.1 Number of patches

**Fig. 7:**
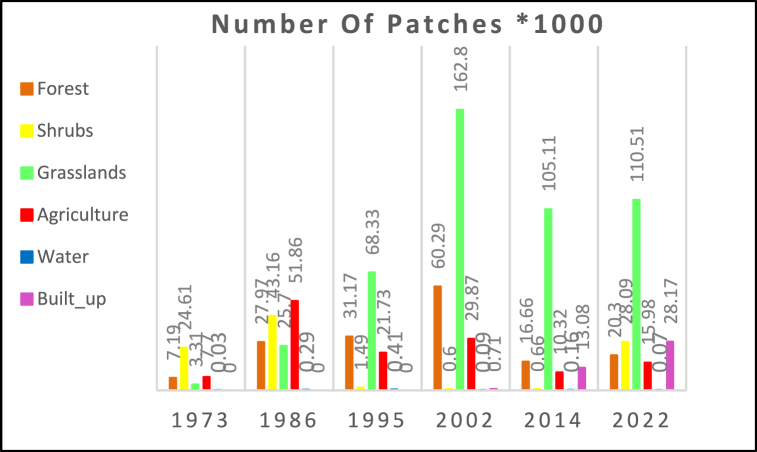
Number of patches.

Landscape metrics analysis in this study area revealed that KRSB is characterized by increases in patch numbers [PN] and patch density for forests, grasslands, and water for the period 1973–2002 and a decrease for almost LULC classes apart from built-up areas between 2002 and 2022 [Fig. 7]. These occurrences ultimately led to attrition [complete disappearance of patches] in some areas and replacements in others, particularly forest lands, shrubs, and grasslands. From 1973 to 2002, forests and grasslands showed an increase in PN by +738 %, and +4818.4 % respectively while water showed a similar trend of +1267.7% between 1973 and 1995. Similarly, built-up areas show an increment of +3867% since its detection in 2002 and 2024. Overall, a decline in PN was observed between 2002 and 2014 apart from built-up areas. Especially, forests, grasslands, and agriculture PN values decreased by −72.3%, −35.6%, and −65.5 over the same period respectively. However, a new trend of further PN increases was observed between 2014 and 2022 [[34], [35]]. highlights that a high number of patches of habitat was directly proportional to the levels of fragmentation.

Despite urbanization occurring since 1973, this phenomenon couldn't be detected due to the coarse spatial resolution of the Landsat sensors available at that time. This limitation was exacerbated by the mixed spectral signatures between rooftops (including grass-thatched and tiled roofs) and the surrounding shrub covers. Consequently, grassland emerged as the most vulnerable and threatened habitat. The increase in its productivity numbers is significantly higher compared to forests, primarily because many grassland patches were directly converted into farmlands. In contrast, forests, in some cases, underwent an intermediate stage of conversion into shrublands before being transformed into farmlands or grasslands. This key finding is in agreement with the result reported by Ref. [65]. Despite agriculture showing low productivity numbers, land has been extensively divided among families under land tenure laws. However, satellite sensors are unable to distinguish one parcel of land belonging to one family from another due to spatial resolution limitations. Consequently, the sensor considers most parcels as one large parcel.

#### 3.2.2 Interspersion and juxtaposition index [IJI]

**Fig. 8:**
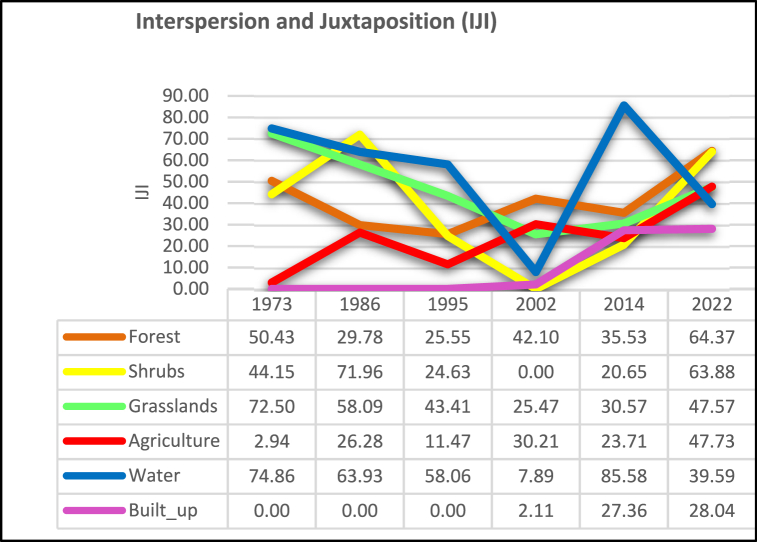
Interspersion and juxtaposition.

IJI varies inconsistently over time across all the LULC classes [Fig. 8]. The high variation is an indication of a high, random, and unpredictable level of landscape fragmentation. The built-up area class shows an increasing IJI which shows its growth in terms of evenness and dispersion. The built-up area has increased IJI values by +1227% from 2002 to 2022. Agriculture also shows a continuous growth of IJI as more farmland patches get interconnected.

For example, IJI for agriculture increased by +1522 % for the study period 1973–2022 while IJI values for grasslands declined by - 34.3% over the same study period. However, forests, shrubs, and water show an inconsistent pattern as most deforestation occurs differently across the landscape and whereas some forests are first reduced to shrubs, some are directly converted to bare lands or agricultural lands. However, a decline in grasslands occurs at the expense of increasing the evenness of agriculture as it is easily convertible.

#### 3.2.3 Edge density

**Fig. 9:**
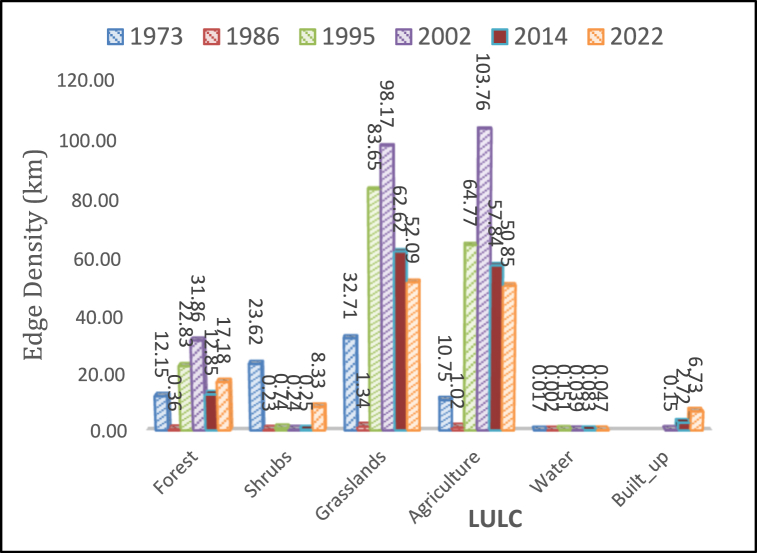
Edge density.

Edge density across all land covers was inconsistent [Fig. 9]. ED decreased in forests, grasslands, shrubs, agriculture, and water especially for the period 1973 to 1986 before increasing again from 1986 to peak in 2002.

The year 2002 marked significant ED values, with grasslands registering 98.2 and agriculture reaching 103.8. Over the span from 1986 to 2002, remarkable percentage increases in ED were observed across various land cover types: agriculture surged by +10086%, grasslands by +7240%, shrubs by +1.63%, forests by +8668.8%, and water by +9933.3%. However, a contrasting trend emerged between 2002 and 2022, showcasing a notable decline in ED. Agriculture witnessed a reduction by −51%, while grasslands experienced a decrease of −46.08%. Notably, forests displayed a slight increase during the period from 2014 to 2022, attributed to fragmented patches and encroachment, particularly evident around Mt. Elgon National Park. Encroachment by residents, often engaging in concealed farming, has led to the fragmentation of forest edges.

Conversely, the built-up area exhibited a continuous surge, escalating by +4372.2% from 2002 to 2022. This growth is expected to intensify, especially in proximity to protected forests like those near Mt. Elgon, as settlements continue encroaching upon these vital biomes. Similarly, the expansion of agriculture has encroached upon grasslands, mirroring the trend observed in ED values over time. These trends underscore the complex interplay between human activities and ecological systems, highlighting the urgent need for sustainable land management practices and conservation efforts to mitigate adverse impacts on ecosystems and biodiversity. As emphasized by Ref. [45], variations of ED indicate a major change and reduction of the spatial heterogeneity scale of the landscape. This was true in the grassland class. Likewise, the higher value of ED indicated by the forests shows no or little central tendency of the ecosystem as a result of some invasions and disturbances ich have been detected across many landscapes [92].

#### 3.2.4 Connectiveness

Connectiveness measures the degree to which various landscape features, such as habitat patches or land cover types, are connected or fragmented. It assesses how easily ecological processes or species can travel across the terrain. Maintaining biodiversity, promoting gene flow, and bolstering environmental resilience all depend on connectiveness. Connectivity measures usually take into account elements like patch size and closeness, as well as the existence of passageways or other elements that make it easier to traverse between patches. In order to maintain or restore ecological connection, conservation and land management actions can be influenced by these measures, which aid in evaluating a landscape's overall structural connectivity [93].

**Fig. 10:**
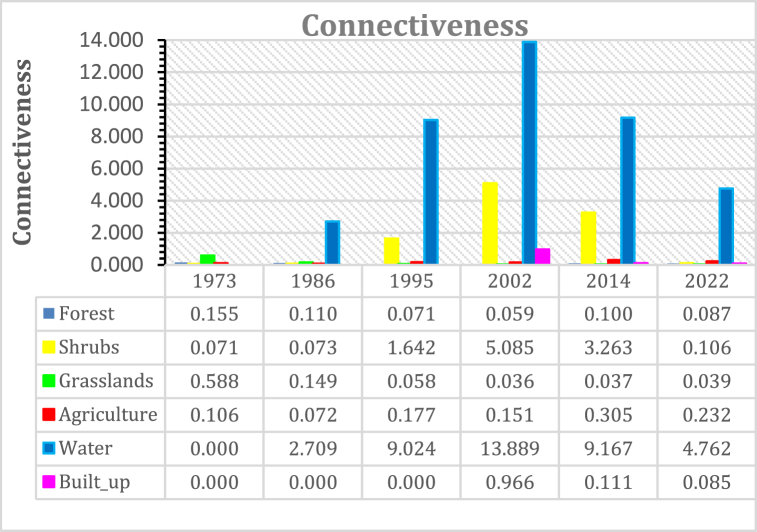
Connectiveness.

All classes show an increase in connectedness between 1973 and 2002 and built-up areas from 2002 [Fig. 10]. Water and shrubs show connectiveness change of +13.88, +5.014 respectively for the period 1973 to 2002 and urban at +0.966 for the period 1995–2002.

The same classes show a decline between 2002 and 2022 possibly as a number of their patches and the distance between them widened.

#### 3.2.5 PAFRAC

**Fig. 11:**
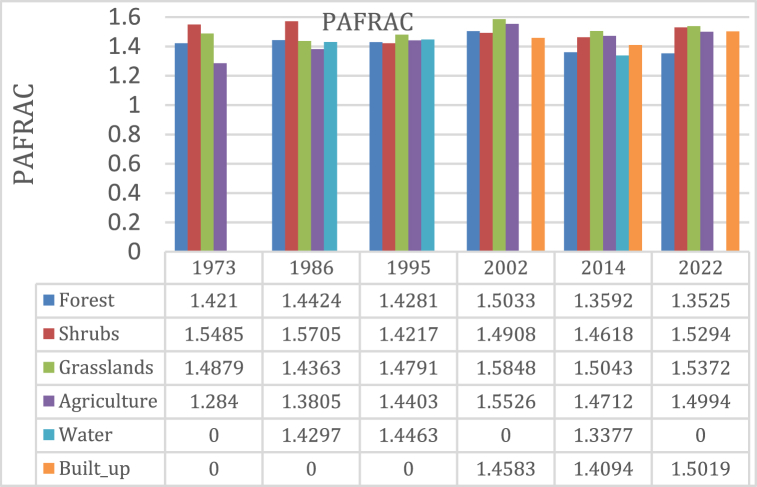
Pafrac.

The analysis of PAFRAC values [Fig. 11] reveals distinct trends over different time periods. Initially, from 1973 to 1995, there is an upward trajectory in PAFRAC values, followed by another increase between 1995 and 2002. However, from 2002 to 2014, PAFRAC values exhibit a decline. It's important to note that the trend for water shows irregular fluctuations due to the methodology employed in calculating PAFRAC within the FRAGSTAT tool. PAFRAC values are sensitive to changes in patch sizes and forms across different land cover classes. Specifically, the value remains constant if the form remains unchanged, and it returns as ‘N/A′ when patches of any specified class are less than 10. PAFRAC values range from 1 to 2, indicating the complexity of patch shapes. A value of 1 suggests simpler patch shapes, while a value approaching 2 indicates highly irregular shapes. This underscores the nuanced nature of landscape fragmentation, influenced by various factors including land use changes and patch configurations. Understanding these trends is crucial for effective landscape management and conservation efforts [26].

As shown in Figure [14], all patches of different classes have changed in form an indication of continuous human disturbances that have led to attritions or replacements of some patches with patches of different classes.

#### 3.2.6 Core area

**Fig. 12:**
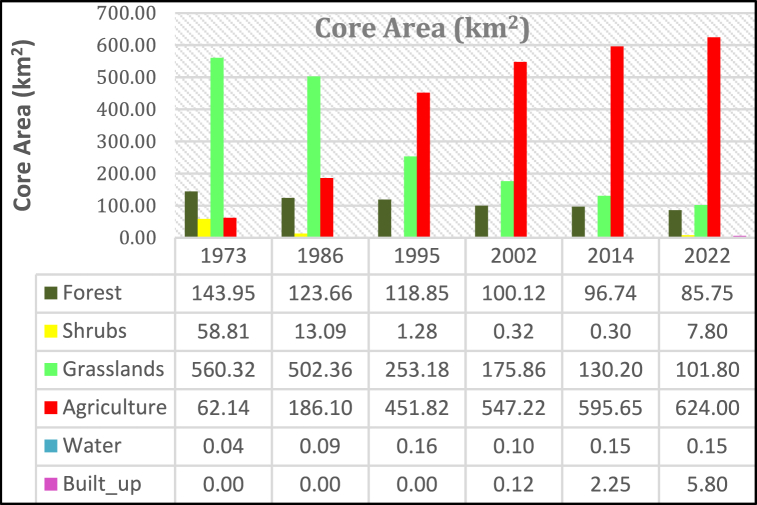
Core area.

The core area is an area of a land cover class further from a defined edge distance [45]. In this study, 100 m was defined. The core area of agriculture, built-up area, and water shows an increase over the study period while grasslands, forests, and shrubs show a decline over the same period [Fig. 12]. For the entire period, the core area of grasslands, forest, and shrubs show a decline of 81.83%, 40.4%, and 86.73% respectively for the entire study period.

However, agriculture and urban growth show an increase of +9042% and +4567% for the period 1973–2022 and 2002–2022 respectively. A reduction of grasslands corresponds with an increasing core area for agriculture. It is critical that forests and grasslands which serve the function of promoting infiltration in watersheds on a continuous decline.

### 3.3 The extent of landscape fragmentation at the patch level

#### 3.3.1 Maximum patch area

**Fig. 13:**
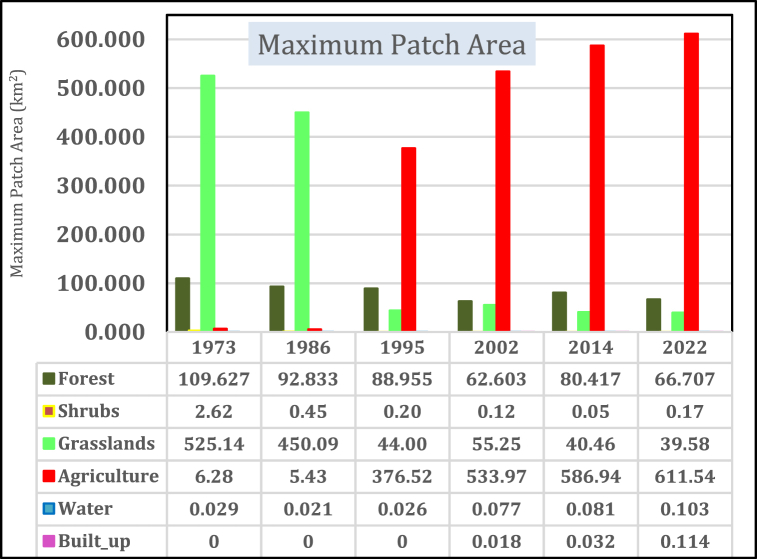
Maximum patch area.

The maximum patch area [Fig. 13] depicts a growing patch of land cover in a landscape. The general trend for the study period 1973–2022 shows an increasing trend for agriculture, built-up areas, and water and a decreasing maximum patch area for grasslands and shrubs. Agriculture has expanded by 605.26 km^2^ (9634.86%) for the period 1973–2022 and built-up area by 0.0963 km^2^ (535%) for the period 2002–2022 while water increased by 0.0738 km^2^ (256%) with the highest being experienced between 1995 and 2002 by 0.0513 km^2^ (196.6%). Forests have reduced by 42.91 km^2^ [60.86%] for the period 1973–2022 while shrubs reduced by 2.57 km^2^ [98.1%] for the period 1973 to 2014 before they increased slightly between 2014 and 2022 by 0.121 km^2^ (243%) between 2014 and 2022.

The agriculture patch area appears larger due to the increased division of land among different farmers, rendering them indistinguishable by satellites with limited 30 m spatial resolution. Water exhibits variations owing to seasonal changes and the extent of exposure of existing wetlands to continuous encroachment and human activities. The results of an expansive large patch area coincide with the findings by Ref. [80] in the Mara basin.

#### 3.3.2 Maximum patch ENN

**Fig. 14:**
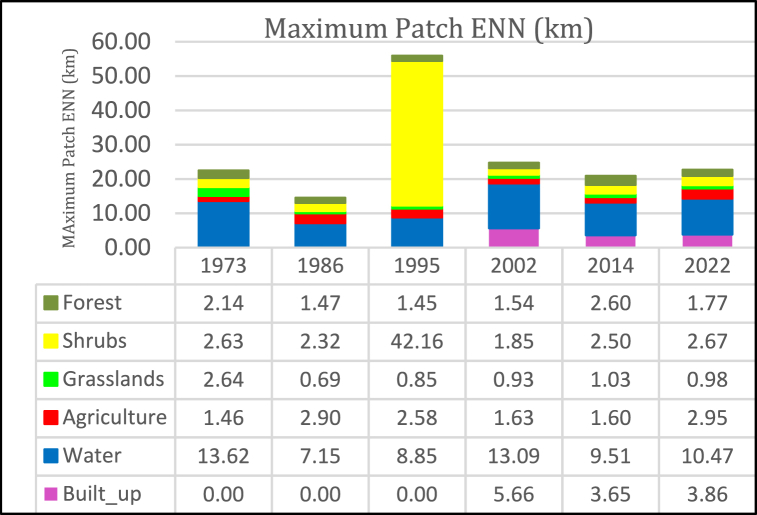
Maximum patch ENN.

The ENN [Fig. 14] depicts the distance between patches of the same LULC class. The higher the distance, the higher the separation. For KRSB, the ENN for all LULC classes shows a non-uniform separation distance.

For instance, shrubs exhibited the highest separation observed during the period 1996–1995, reaching 39.8 km. This period marked significant fragmentation and separation of shrubland. Water, on the other hand, showed substantial variation, with a major Euclidean Nearest Neighbor (ENN) variation between 1973 and 1986 (−6.464 km) and a general decline of 4.11 km over the study period from 1973 to 2022. Agriculture patches displayed relatively constant values, reflecting the interconnectedness of farms owned by various agencies and residents. Similarly, the maximum forest ENN was recorded at 2.13 km in 1973 and increased to 2.6 km in 2014. The high maximum forest patch ENN in 2014 indicates forest decline and the elimination of some existing patches. In contrast, the built-up area's maximum patch ENN peaked in 2002 at 5.66 km upon its initial detection but decreased to 3.86 km by 2022. This reduction is attributed to the decreasing distance resulting from the continuous expansion of built-up areas due to urban sprawl and human settlements.

#### 3.3.3 Maximum patch CONTAGION and FRAC

**Fig. 15:**
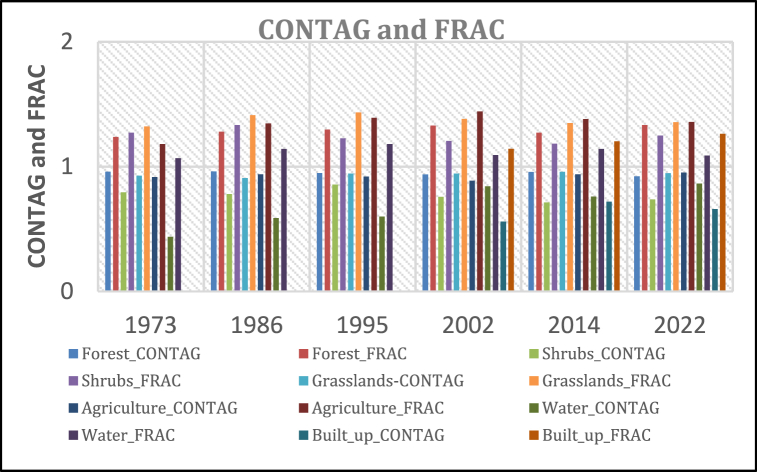
Maximum contag and FRAC

Overall, maximum contagion in KRSB [Fig. 15] varies across all LULC classes with the forest being the highest between 1973 and 1995 and the built-up area being the lowest since its detection between 2002 and 2022.

FRAC explains the patch complexity of different land covers over time and relates the perimeter of a patch to a patch area. This indicates that a change in the size of a patch without changing the form of a patch does not change the index and the value varies between 1 and 2. For KRSB [Fig. 15], it was observed that all land cover changed the form a sign that all land covers are fragmented.

Low contagion values indicate high dispersion and this is observed for water whose detection is spatially distributed across the watershed. The built-up area is also spatially distributed in the watershed and thus has low contagion values even though this rose between 2002 and 2014 by +28.4 %. This is reflected by Ref. [94] who highlights that high contagion indicates low dispersion because patches are close to each other. Even though the value for grass seems high it only indicates that the remaining patches are adjacent to each other and confined in key areas such as the riparian corridors.

The analysis of FRAC values unveils significant shifts in landscape fragmentation, notably between grasslands and agriculture over distinct time intervals. Grasslands exhibited the highest FRAC between 1973 and 1995, with a value of −0.1113. However, this dominance was overtaken by agriculture between 2002 and 2022, with a FRAC of 0.0831. This transition coincides with land use and land cover (LULC) changes, where extensive conversion of grasslands to agriculture occurred due to their vulnerability and ease of conversion. In contrast, forests display a more moderate change in FRAC and form due to the presence of large forest patches within protected areas. Initially, there was a notable increase in FRAC by 0.0909 (+7.3%) between 1973 and 2002. However, this trend reversed between 2002 and 2014, with a decrease of −4.3%, before experiencing a subsequent increase between 2014 and 2022 by +4.85%. Water areas consistently show the lowest FRAC values, reflecting their minimal coverage within the KRSB region and low susceptibility to changes in form. This stability in water coverage underscores its importance as a relatively unaltered component of the landscape. These trends highlight the dynamic interplay between land cover changes, FRAC values, and landscape fragmentation. Understanding these dynamics is essential for effective land management strategies aimed at preserving ecosystem integrity and biodiversity within the KRSB region [94]. indicates that high FRAC values indicate high fragmentation levels in terms of form and size and this leaves the KRSB vulnerable to environmental issues that could affect the watershed health and ecosystem functioning.

### 3.4 The extent of landscape fragmentation at the landscape level

#### 3.4.1 Edge density, contagion and IJI (%)

**Fig. 16:**
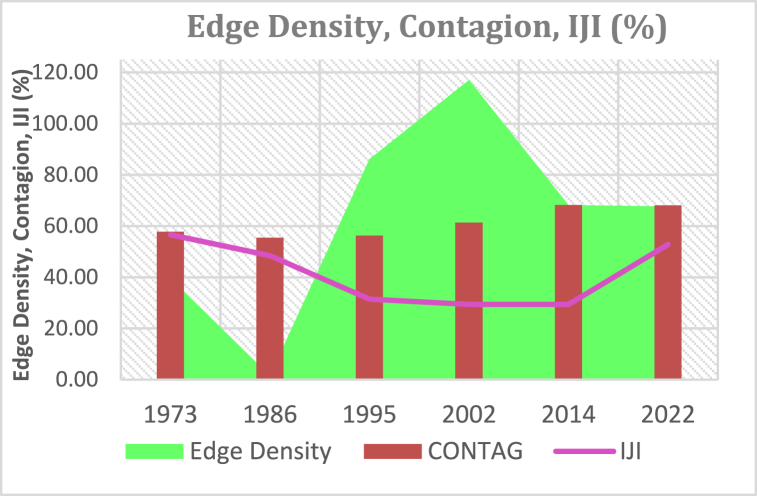
Edge density, contagion and IJI (%).

As shown in Fig. 16, Edge density for KRSB shows a general decrease between 1973 and 1986, a rise for the period 1986–2002, and a decline between 2002 and 2022. Contagion which is a key metric measures dispersion and includes interspersion between patches.

The ED of the KRSB landscape (Fig. 16) shows a decreasing trend from 1973 to 1986 and rose between 1986 and 2002 by +115.63 when more forests, shrubs, and grasslands were reduced at the expense of increasing agricultural land and built-up area. This signifies the period when more edges were generated due to a surge in human settlements and encroachment of the protected areas. A decrease between 2002 and 2014 (−49.49%) as the remaining patches were eliminated or replaced by farmlands and built-up areas. According to Ref. [[13], [94]], edge density refers to the number of total edges in the total landscape area, and the higher the number of edges, the higher the level of landscape structural fragmentation.

The CONTAGION analysis shows a continuously increasing contagion for the period 1973–2022 (+10.30) which shows a continuous dispersion of patches especially for forests, shrubs, grasslands, and water. This scenario indicates that a once integrated ecosystem fabric of the landscape has been fragmenting leaving the watershed vulnerable to erosion and flooding. The disconnected landscape further reduces infiltration which results in acerated runoff [95].

**Fig. 17:**
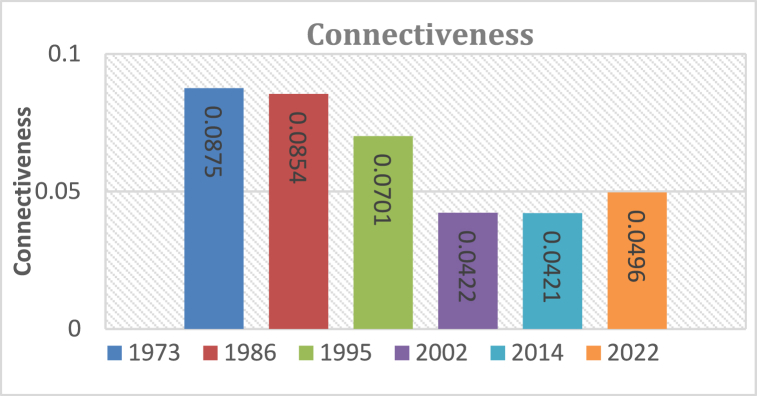
Connectiveness.

Whereas, CONTAGION increases over time, IJI for KRSB (Fig. 16) shows a decrease between 1973 and 2014 by −27.2 % and an increase of +23.4% between 2014 and 2022. The decrease shows the closeness of fragmented patches of different land cover classes while between 2014 and 2022 exhibited an increase in dispersion of different land covers [Fig. 17]. This is due to the emergence of built-up areas and extended agricultural lands which has dispersed other classes even more [34]. found similar results where they indicated that an increase in IJI values indicates a high magnitude fragmentation and hence the landscape experienced the worst interspersion between 2014 and 2022 a trend that needs to be considered for urban planning and human settlements.

#### 3.4.2 Connectiveness

Connectivity in KRSB has been reducing over time from 1973 to 2014 [−0.0454] and a slight increase between 2014 and 2022 (+0.0496). The increase [Fig. 17] is associated with reforestation efforts by individual farmers but as observed from the field, reforested patches serve short-term functionalities as they are harvested for commercial purposes. Further, the increase of built-up areas trail has a significant effect on the increases in connectiveness of patches of different land classes even though the general trend is a decrease leading to ecoscapes which minimize biological diversities, structural functionalities necessary to improve hydrological performances of the watersheds.

According to Refs. [93,94], any changes in connectiveness lead to a reduction in landscape structural functioning, habitat loss, and biodiversity loss necessary to promote climate change resilience of not only wildlife, and vulnerable plant species but also ecosystem functioning of the landscape within the natural limits.

#### 3.4.3 Shannon Diversity Index and Shannon Evenness Index

**Fig. 18:**
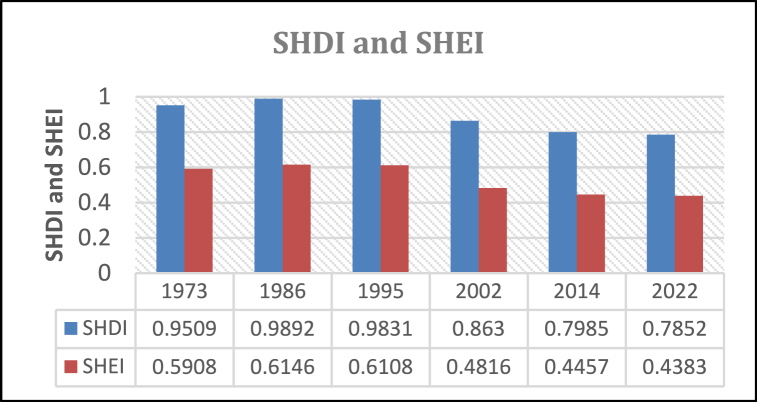
Shannon diversity and evenness index.

Both SHDI and SHEI show a general declining trend over the study period. The rates stand at −17.42% and −25.8% respectively and this indicates a continuous habitat loss and replacement of total elimination of patches of different classes in the landscape [Fig. 18].

The decrease in SHDI shows a decrease in the intermixing of patches of different classes in the entire KRSB landscape which corresponds with a decrease in evenness of species of different classes. This is an indication of high fragmentation in terms of the richness of land cover species and adjacencies. A decrease in both SHDI and SHE signifies a degraded landscape whose ecosystem functionality is compromised. This leaves most areas vulnerable to unprecedented environmental changes such as increased runoff, soil losses, and compromised biomes. Relationship between SHDI and SHEI and flood peaks have been applied by Ref. [71] and has found that there was a positive correlation even though this needs to be established in different landscapes.

### 3.5 Driving forces of landscape fragmentation

Landscape change in KRSB is driven by several factors. Based on the feedback received from 32 survey questionnaires, and oral interviews, hotspot areas vulnerable to landscape degradation were first analyzed followed by the nature of prevalent LULC conversion and the main drivers of landscape fragmentation. Results are discussed in sections 3.5, 3.5.1.2.

#### 3.5.1 Identified hotspot areas vulnerable to landscape fragmentation

**Fig. 19:**
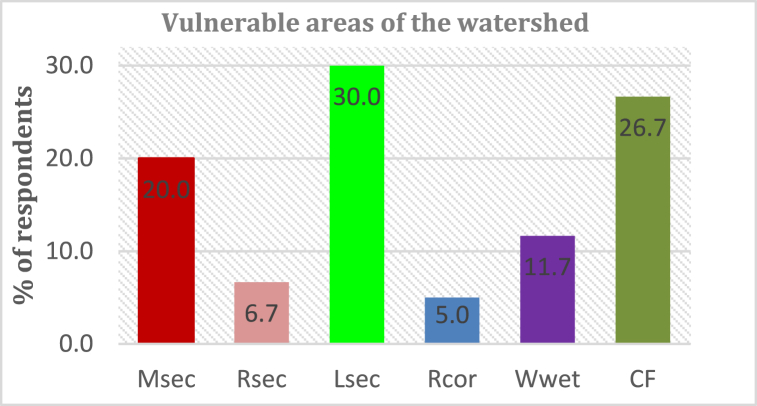
Vulnerable areas of the watershed.

Vulnerable areas of the watershed and the nature of land cover conversion were assessed using survey questionnaires and oral interviews. Results are presented in Fig. 19.

M_sec_ = Middle section [Endebess, Kaisagat & Zea areas], R_sec_ = Recharge sections [Mt. Elgon], L_sec_ = Low recharge areas [Kitale town, Sibanga, Kibomet], R_cor_ = Riparian corridors, W_wet_ = Wetlands and C_F_ = Commercial farms. From responses, it is observed that all sections are vulnerable even though lower sections were highlighted as being vulnerable. This is due to frequent flooding and high sediment load that has become a common menace in the downstream areas. Further, most of the wetlands have been encroached or depleted.

#### 3.5.2 Nature of LULC conversion insights

**Fig. 20:**
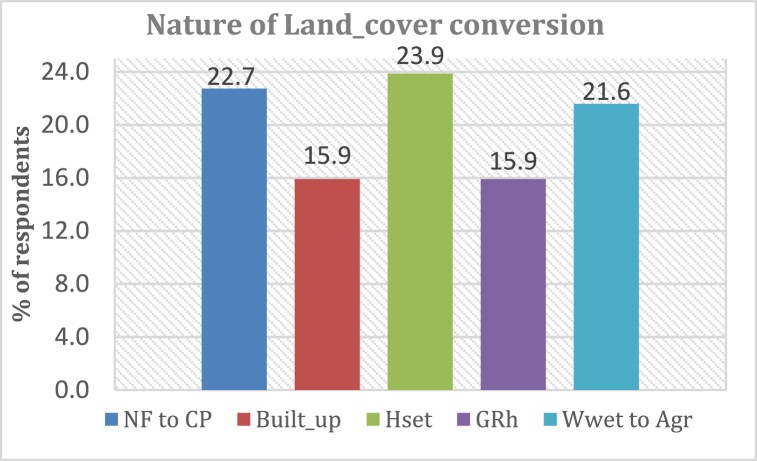
Nature of land-cover conversion NF = Native forests, CP = Croplands, H_**set**_ = Human settlements, G_**Rh**_ = Greenhouses and W_**wet**_ = Wetlands.

Inhabitant's views indicated a myriad of LULC conversion scenarios in KRSB. The findings [Fig. 20] indicated emergency of large-scale greenhouse farms whose effect on the landscape structure was noted by the respondents.

The watershed is dominated by the conversion of various classes such as shrubs, grasslands, and forests to human settlements, the conversion of wetlands to agricultural land [maize and sugarcane], and built-up areas [mostly urban settlements and infrastructure]. A new trend was also observed from the feedback where the number of greenhouses is increasing and this may trigger direct transformation of rainfall to runoff. This observation concurs with findings by Ref. [75], [96]

#### 3.5.3 Driving forces of landscape fragmentation in KRSB

One of the main causes identified was farmers' adoption of an unsustainable economic model, leading to overcultivation of their land in pursuit of maximizing profits. This practice was acknowledged by farmers across different regions of the watershed, resulting in soil degradation and increased susceptibility to erosion, hindering vegetative regeneration.

Ineffective land-use management laws were also highlighted as contributing factors. Despite legal protections for riparian corridors, it was observed that the Koitobos River and its distributaries were extensively encroached upon, with little to no forest remnants left. Farmlands have largely replaced riparian zones in these areas.

Oral interviews revealed a significant issue concerning the alleged bribery of individuals posing as law enforcement officers to facilitate the destruction of forest boundaries and expansion of farmlands, particularly along the protected forests surrounding Mount Elgon National Park. Some small farms were even concealed within the main forest habitat. Major crops grown in these encroached regions included millet, maize, sorghum, and Irish potatoes.

Politics and power dynamics, occurring on a five-year cycle, were identified as another important driving force behind landscape fragmentation. Campaign promises of farming flexibility made by politicians have contributed to sporadic incursions into forest ecosystems, prompting discussions about forest border locations. Furthermore, the establishment of new satellite urban centers, spurred by devolution, has led to significant population growth within the watershed. This population boom has intensified farming practices and competition for space along riparian corridors, both in urban and rural areas.

Rapid urbanization has also increased demand for wood products, resulting in heightened logging activities, including illegal logging in protected ecosystems like Mount Elgon. Insecurity concerns have led to the removal of a nearby forest known as the Mt. Elgon Academy Forest, which has been utilized as a crime scene, including for the disposal of human remains. As a result, much of the forest has been converted into farmlands, cultivating vegetables, beans, and Irish potatoes.

Additionally, human-wildlife conflict, particularly in former wetland areas, has driven landscape changes. Encroachment and destruction of wetlands, such as Kitalale, Sikubu Dam, and Chemususu, have occurred due to past regular attacks on cattle by wildlife, notably pythons. However, these instances have become increasingly rare as most wetlands have been destroyed.

#### 3.5.4 Implication for landscape conservation and restoration

Over the last 49 years, rates of landscape fragmentation in KRSB have been augmented. Forests, grasslands, and shrubs show high fragmentation rates based on high increments of PN, ED, FRAC, and a decline in SHDI, SHEI, and IJI and this threat will possibly lead to habitat loss, and changes in hydrological processes as habitat fragmentation is a precondition of habitat loss and ecosystem dysfunction. According to Refs. [97,98], habitat fragmentation, ecosystem dysfunction, and habitat loss go hand in hand and outcomes lead to a compromised landscape whose processes are beyond the normal range.

These changes point to a failing landscape matrix that is marked by a decline in habitat diversity and connectedness, particularly as agricultural and urban areas grow. Analyses also revealed a declining AREA_MN and COA, indicating that unless sustainable watershed conservation measures are implemented, core-dependent ecospecies are likely to face extinction or increased survival strain.

Natural ecosystems in the form of fragmented forest areas and watersheds give residing communities access to fuel, wild fruits, food, medicine, and lumber. Communities close to the Mt. Elgon National Park play a crucial role in preserving the park's forests and delicate ecosystems.

To provide ecological services including regulating climate, nourishing the Koitobos River's flow regime, purifying the air, preventing soil erosion, and controlling soil fertility loss, this portion of the watershed is essential.

It is also crucial that a rise in populated areas drives increasing demand for food and wood goods. Farmers will be under pressure to increase agricultural output as a result, which can also entail having full access to the already-degraded riparian zones. Governmental organizations should monitor the regeneration, conservation, and protection of riparian zones to stop this. This should be done in tandem with providing KRSB inhabitants with civic education, with a focus on the value of citizen involvement in watershed management.

Furthermore, to prevent instances of free-range encroachment, it is crucial to precisely define the boundary limitations of Mt. Elgon National Park. Additionally, it is important to reassess institutional fragmentation as well as the overlap of tasks and responsibilities for the protection of important ecosystems. Rehabilitating damaged wetlands is also crucial, as respondents have mentioned them as excellent providers of ecosystem services. Finally, it is empirically proven that planted forest sections on individual farms only persist for a certain amount of time before they are taken for lumber, even with some individual replanting initiatives. Promoting a fee for ecosystem services is one alternative; this was considered a workable concept during the interviews.

## 4 Conclusion

The study amalgamated LULC change, landscape structural, and pattern analyses to assess the degree of fragmentation in the study area over four decades, from 1973 to 2022. Findings highlighted significant spatial landscape transformations and alterations in land cover structure, particularly in the KRSB region, which was identified as highly fragmented. This fragmentation was evidenced by substantial changes in key metrics such as PN (from 4284 to 20312), Connectiveness (from 0.0875 to 0.0496), and SHDI (from 0.9509 to 0.7852) between 1973 and 2022. The study revealed a consistent increase in fragmentation at class, patch, and landscape levels over time, underscoring the urgent need for ecological restoration and integrated watershed management to mitigate potential adverse impacts on hydrological health. The observed negative effects of urbanization and human settlement, coupled with ineffective forest plantation practices primarily driven by commercial interests, jeopardize numerous nature-based ecosystem services. Addressing these challenges necessitates comprehensive and sustainable environmental restoration and management strategies, with due consideration to traditional beliefs and indigenous knowledge. Regulatory frameworks promoting sustainable land-use practices are imperative to achieve ecological and socioeconomic well-being in the research region. The study's findings offer valuable insights for watershed managers and policymakers, facilitating informed decision-making for maintaining a healthy watershed. Future research endeavors should focus on developing spatial frameworks for landscape restoration and assessing the implications of landscape structural changes on ecosystem services.

The temporal scope of this study spans four decades and hence short-term variations or recent developments may have been overlooked and yet could influence the landscape fragmentation. The Landsat imagery used in this study is limited by its spatial resolution which could leave out some finer grains spatial patterns or localized impacts limiting its applicability to smaller scale management interventions. Similarly, certain assumptions underlying analytical methods employed in the study could introduce uncertainties. In the same spectrum, while the study acknowledges the importance of traditional knowledge and regulatory frameworks, deeper exploration of socio-economic dynamics shaping land-use decisions could provide additional insights. Lastly, findings of this research are specific to the study area and may not be transferable to other watersheds without considering contextual differences. Based on these limitations, the authors would like to recommend future research as follows; Longitudinal studies should be conducted to track landscape dynamics over shorter time intervals to capture recent changes and their implications on ecosystem functioning. Data enhancement is required by investing in improving data quality and coverage to leverage advanced remote sensing techniques and ground truthing to enhance accuracies of the analyses. Multiscale analysis is also required to integrate fine grained spatial patterns and understand how landscape fragmentation operates across different scales. There is also need to incorporate interdisciplinary perspectives including socials sciences and economics to better understand the human dimensions influencing landscape dynamics. Lastly, stakeholder engagement is required to foster collaboration with local communities to allow communities toco-develop contextually relevant management landscape reconfiguration strategies.

## Funding statement

The authors acknowledge the financial support from the African Union and Pan African University-Institute for Basic Sciences Technology and Innovation

## Additional information

No additional information is available for this paper.

## Data availability statement

Data is available at https://drive.google.com/drive/folders/1NDp5qVKkChtEF7r7ngdP8Ta0Elg0Y-f6?usp=sharing.

## CRediT authorship contribution statement

**Kennedy Wekesa Murunga:** Writing – review & editing, Writing – original draft, Methodology, Investigation, Formal analysis, Data curation, Conceptualization. **Maurice Nyadawa:** Writing – review & editing, Supervision, Project administration. **Joseph Sang:** Writing – review & editing, Supervision, Project administration. **Charles Cheruiyot:** Writing – review & editing, Supervision, Project administration.

## Declaration of competing interest

The authors declare the following financial interests/personal relationships which may be considered as potential competing interests: Kennedy Wekesa Murunga reports financial support, administrative support, and travel were provided by Pan African University Institute for Basic Sciences Technology and Innovation. If there are other authors, they declare that they have no known competing financial interests or personal relationships that could have appeared to influence the work reported in this paper.
